# Ultrasound-guided cryoablation of early breast cancer: safety, technical efficacy, patients’ satisfaction, and outcome prediction with MRI/CEM: a pilot case-control study

**DOI:** 10.1186/s41747-024-00515-4

**Published:** 2024-10-22

**Authors:** Francesca Galati, Marcella Pasculli, Roberto Maroncelli, Veronica Rizzo, Giuliana Moffa, Bruna Cerbelli, Giulia d’Amati, Carlo Catalano, Federica Pediconi

**Affiliations:** 1 Department of Radiological, Oncological and Pathological Sciences, Sapienza—University of Rome, Rome, Italy; 2 Department of Medical and Surgical Sciences Biotechnology, Sapienza—University of Rome, Rome, Italy

**Keywords:** Breast neoplasms, Contrast-enhanced mammography, Cryosurgery, Magnetic resonance imaging, Patient satisfaction

## Abstract

**Background:**

This pilot prospective study aimed to evaluate ultrasound-guided cryoablation of breast cancer (BC) by assessing: (i) technical efficacy as the presence of necrosis in surgical specimens and rate of complete tumor ablation; (ii) safety as incidence and severity of complications; and (iii) patients’ satisfaction using a dedicated questionnaire. In addition, (iv) we tested the capability of magnetic resonance imaging (MRI) or contrast-enhanced mammography (CEM) to predict cryoablation efficacy.

**Methods:**

From 07/2022 to 01/2023, we enrolled 20 patients with early-stage BC scheduled for breast surgery. Ten of them, with a cryo-feasible cancer location, were sent to cryoablation (cryo-group) and ten to routine surgical practice (control group). Both groups underwent surgery and were asked to answer a satisfaction questionnaire.

**Results:**

Of eleven patients screened for cryoablation, only one refused to be treated at another hospital (acceptance rate 10/11, 91%). Surgery was quadrantectomy in 19 cases and mastectomy in 1. In the cryo-group, the procedure was completed and steatonecrosis was observed in 10/10 cases, with complete tumor ablation in nine of them. The post-procedural status was evaluated with MRI in five patients, with CEM in four patients, and with ultrasound in one patient who refused MRI and CEM. MRI or CEM correctly predicted complete cryoablation in eight patients and incomplete cryoablation in one patient. Patients in both groups did not have serious complications and responded positively to satisfaction questionnaires.

**Conclusion:**

Ultrasound-guided cryoablation of early-stage BC is well accepted by patients, effective, and safe. MRI and CEM were able to predict the procedure's technical efficacy.

**Trial registration:**

https://clinicaltrials.gov/study/NCT05727813 updated February 14, 2023.

**Relevance statement:**

Our pilot study showed that ultrasound-guided cryoablation is a promising nonsurgical alternative for treating early-stage BC.

**Key Points:**

- Ultrasound-guided cryoablation was effective and safe in early BC patients.
- The procedure was well-tolerated, with low morbidity and high patient satisfaction.
- MRI and CEM predicted cryoablation efficacy, in accordance with histopathologic findings.
- Cryoablation can be considered a potential alternative to surgery in selected patients.

**Graphical Abstract:**

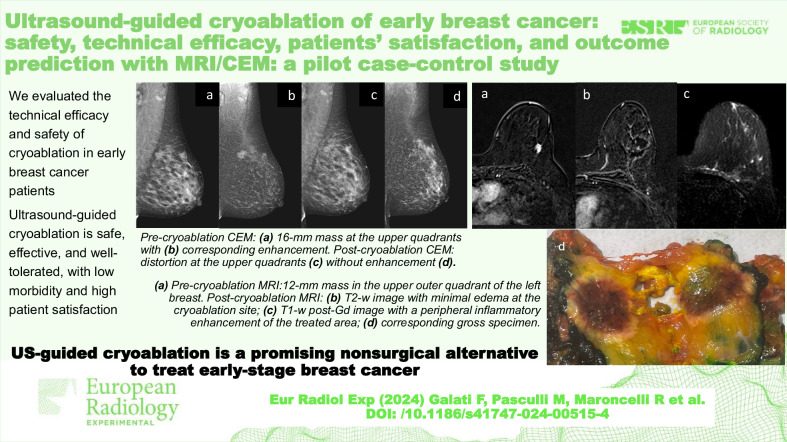

## Background

Breast cancer (BC) is the most frequent malignant tumor in the female sex [1]. As the accuracy of screening programs continues to improve, a steady increase in the incidence of early BC and a parallel decrease in mortality have been observed in recent years [1]. With the capability to detect lesions at an earlier stage, the standard of care for BC treatment has evolved over the years from mastectomy to conservative surgery to reduce morbidity and improve quality of life, while maintaining therapeutic effectiveness [2].

Although breast-conserving surgery, including local excision and quadrantectomy, is generally accepted as the preferred alternative to mastectomy for tumors up to 3 cm in size [3], common postoperative complications include seroma, infection, hematoma, swelling, persistent postoperative pain, and fat necrosis [4]. In this context, minimally invasive treatments could be considered as an interesting alternative option for BC treatment to avoid surgery-related complications, optimize the cosmetic results, and reduce patient discomfort and psychological distress. Moreover, minimal or noninvasive ablation could be an option for patients who are not eligible for surgery [3]. Minimally invasive imaging-guided treatments include radiofrequency, microwave, laser ablation, cryoablation, and high-intensity focused ultrasound.

Cryoablation is a minimally invasive procedure that uses freezing and thawing cycles to induce necrosis of the targeted tissue, while preserving the surrounding tissue architecture [5]. This procedure has been successfully used for the ablation of liver, prostate, and kidney tumors, for palliative treatment of locally advanced BCs, and has been approved by the United States Food and Drug Administration (level A evidence) for clinical use in the ablation of breast fibroadenomas [6, 7]. With the exception of cryosurgery, all the above-mentioned techniques use hyperthermia that melts cell membranes and causes protein denaturation [3, 7]. In contrast, cryoablation leaves tumor proteins and tumor-associated antigens intact, with the potential to stimulate an antitumor immune response by increasing the expression of tumor neoantigens specific to tumor cells, which are then attacked and destroyed. By exploiting this effect, cryoablation in combination with immunotherapy may be the key to treating both early-stage BCs and patients who are unfit for surgery [8, 9]. Therefore, this approach is potentially superior to other ablative technologies and may become an additional tool in the oncological treatment of BC.

The primary aim of this prospective pilot case-control study was to evaluate ultrasound-guided cryoablation of BC by assessing: (i) technical efficacy as the presence of necrosis in surgical specimens and rate of complete tumor ablation; (ii) safety as incidence and severity of complications; and (iii) patients’ satisfaction using a dedicated questionnaire. As a secondary endpoint, (iv) we tested the capability of contrast-enhanced magnetic resonance imaging (MRI) or contrast-enhanced mammography (CEM) to predict cryoablation efficacy.

## Methods

### Ethical approval

The study obtained the approval of the Institutional Review Board of “Sapienza” University of Rome (ref. 6528, approved 24.11.2021). This was a pilot case-control, prospective, study on early-stage BC (T1 N0) treated with ultrasound-guided cryoablation, in patients scheduled for breast surgery, and not eligible for neoadjuvant therapy.

### Patient eligibility

Patients aged ≥ 18 years with a single, biopsy-proven, early-stage invasive BC (T1 N0) up to 20 mm in diameter, clearly visible on ultrasound, with a minimum distance of 1.5 cm between the tumor edge and the skin and of 2 cm between the tumor edge and the nipple, not eligible for neoadjuvant therapy, were enrolled to be sent to cryoablation (cryo-group). Patients with early-stage invasive BC of the same characteristics without a cryo-feasible cancer location were enrolled to be sent to routine surgical practice (control group). Exclusion criteria were: histological diagnosis of pure ductal carcinoma *in situ*, lesions with microcalcifications as the only evidence of BC on mammographic imaging, previous history of ipsilateral or contralateral BC, presence of breast implants, contraindications to the use of contrast media, inability to undergo cryoablation treatment, pregnancy, breastfeeding, or recent childbirth.

Both groups were planned to undergo surgery and asked to answer a satisfaction questionnaire.

### Cryoablation procedure

All cryoablation procedures were performed by a radiologist with 20 years of experience in breast interventional radiology (F.P.) using the ICEfx Cryoablation System (Boston Scientific, Marlborough, Massachusetts, USA). The system was equipped with a disposable 17-gauge cryoprobe (ICE SPHERE 1.5, Boston Scientific, Marlborough, Massachusetts, USA), which produces an ice ball with a maximum section size of 33 × 37 mm^2^, and utilizes argon as a cryogen and a resistance heater for thawing. During the procedure, the radiologist was assisted by a bioengineer (V.S.) with specific expertise in the use of the cryoablation system.

**Fig. 1:**
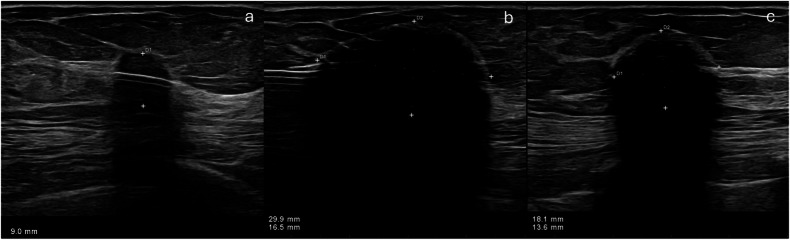
Ultrasound-guided cryoablation of 9-mm cancer in the outer upper quadrant of the left breast. **a** The transversal image represents the cryo-probe inside the core of the target lesion. **b** The transversal image demonstrates the “ice ball” formation, during the first cryoablation freeze cycle, which completely encompasses the tumor. **c** The image shows the reduction in size of the “ice ball” during the first thaw cycle

Cryoablation was performed under ultrasound guidance using a high-resolution unit (Affiniti 70G; Philips, Amsterdam, The Netherlands) with a 12-MHz linear probe. The cryoablation protocol lasted about 25 min: the first freezing cycle (10 min); the thawing cycle (5 min); and the second freezing cycle (10 min). A single cryoprobe was used in all subjects, inserted through a small skin incision after local anesthesia (1% lidocaine) and without patient sedation. Under direct ultrasound observation, the cryoablation freeze–thaw–freeze cycle was performed until both time duration and ice ball diameter goals were achieved (Fig. 1). Ultrasonographic guidance was used to monitor the growth of the ice ball in real time. To prevent frostbite and/or the proximity of the ice front to the skin surface, we injected room-temperature sterile saline between the developing ice ball and overlying skin when this distance became 10 mm or less or when there was visible skin blanching. Finally, the probe was removed, and a bandage was applied.

### Imaging prediction of cryoablation technical efficacy

Cryoablation technical efficacy was evaluated with the same imaging technique (MRI or CEM) before and after the procedure. Patients who had performed MRI or CEM before enrollment in the staging/diagnostic phase, underwent the same type of examination after cryoablation. For the others, the choice of MRI or CEM was based on patient preference or specific contraindications and patient characteristics, such as claustrophobia, obesity, presence of devices not compatible with MRI, or history of adverse reactions to iodinated or gadolinium-based contrast agents.

#### Breast MRI

A breast MRI was performed with the patient in a prone position on a 3-T magnet (Discovery MR 750; GE Healthcare, Chicago, IL, USA) with a dedicated 8-channel breast coil compatible with parallel imaging. The protocol included: axial two-dimensional fast spin-echo T2-weighted fat-suppressed sequence; axial diffusion-weighted echo-planar imaging sequence; axial dynamic three-dimensional spoiled gradient-echo T1-weighted fat-suppressed sequences; and sagittal three-dimensional spoiled gradient-echo post-contrast T1-weighted sequence. Fat suppression of T2-weighted sequences was based on a three-point IDEAL Dixon technique. Diffusion-weighted sequences comprised *b*-values of 0 s/mm^2^, 500 s/mm^2^, and 1,000 s/mm^2^, and corresponding apparent diffusion coefficient maps were automatically calculated. Axial dynamic T1-weighted sequences were performed one time before and nine times after contrast agent administration for a total acquisition time of 363 s. Contrast-enhanced T1-weighted images were acquired after the administration of 0.1 mmol/kg (0.2 mL/kg) of a macrocyclic gadolinium-based contrast agent (Gadoteridol, Prohance; Bracco Imaging, Milan, Italy) at a rate of 3 mL/s. Gadoteridol was power-injected through peripheral venous access (18–20 gauge) and was followed by a 20-mL saline flush. Postprocessing subtraction images were obtained for all examinations. Technical details are summarized in Table 1.

**Table 1:** MRI sequences and technical parameters

Sequence/parameter	Axial 2D T2-weighted fast spin-echo FS	Axial diffusion-weighted echo-planar imaging	Axial 3D gradient-echo T1-weighted FS	Sagittal 3D gradient-echo T1-weighted FS
Repetition time, (ms)	9,000–11,000	4,983–5,314	8	4
Echo time, (ms)	119–120	58	4	2
Flip angle, (degrees)	111	–	15	15
Matrix	512 × 224	150 × 150	512 × 256	224 × 320
Slice thickness, (mm)	3.0–5.0	3.0–5.0	1.4	4.0
Field of view, (mm^2^)	350 × 350	350 × 350	380 × 380	300 × 300
Number of excitations	1	2, 2, and 4	1	1
*b*-values	−	0 s/mm^2^, 500 s/mm^2^, and 1,000 s/mm^2^	−	−
Scan time, (s)	130	230	363	134

*2D* Two-dimensional, *3D* Three-dimensional, *FS* Fat-suppressed

#### CEM

An IMS Giotto Class (IMS Giotto, Bologna, Italy) was the dedicated system used for CEM. A nonionic iodinated contrast agent (Iohexol 350 mgI/mL; Omnipaque 350, GE Healthcare, Chicago, IL, USA) was power-injected through venous access (18–20 gauge) at a dose of 1.5 mL/kg bodyweight at a rate of 3 mL/s, followed by a 20-mL saline flush at the same rate. About two min after the injection, the patient was positioned for mammographic imaging, starting from the affected breast. Two craniocaudal (CC) and mediolateral oblique (MLO) views (early and late acquisition) of both sides were obtained sequentially within 6 min maximum (total time from contrast injection to the last mammogram = 8 min).

Both MRI and CEM examinations were assessed in consensus by two radiologists: Reader 1 (F.P.) and Reader 2 (F.G.), with 20 years and 10 years of experience, respectively. The criteria for reporting the presence of residual tumor were as follows: no residual CEM/MRI enhancement after the procedure was considered successful cryoablation; peripheral inflammatory enhancement of the treated area on CEM/MRI, consistent with edematous imbibition at the site of cryoablation on T2-weighted fat-suppressed images on MRI, was considered successful cryoablation; any focal contrast-enhancement in the peripheral zone or within the tumor bed was considered residual disease and incomplete cryoablation.

### Breast surgery

The preoperative status of the axillary lymph node was always assessed by ultrasound. Within 21 days from enrollment, all patients underwent surgical resection of the primary tumor and axillary sentinel lymph node excision with removal of up to four lymph nodes to ensure oncological safety and achieve an adequate esthetic result. Preoperative localization of BC was wire-guided for all patients.

### Satisfaction questionnaire

**Fig. 2:**
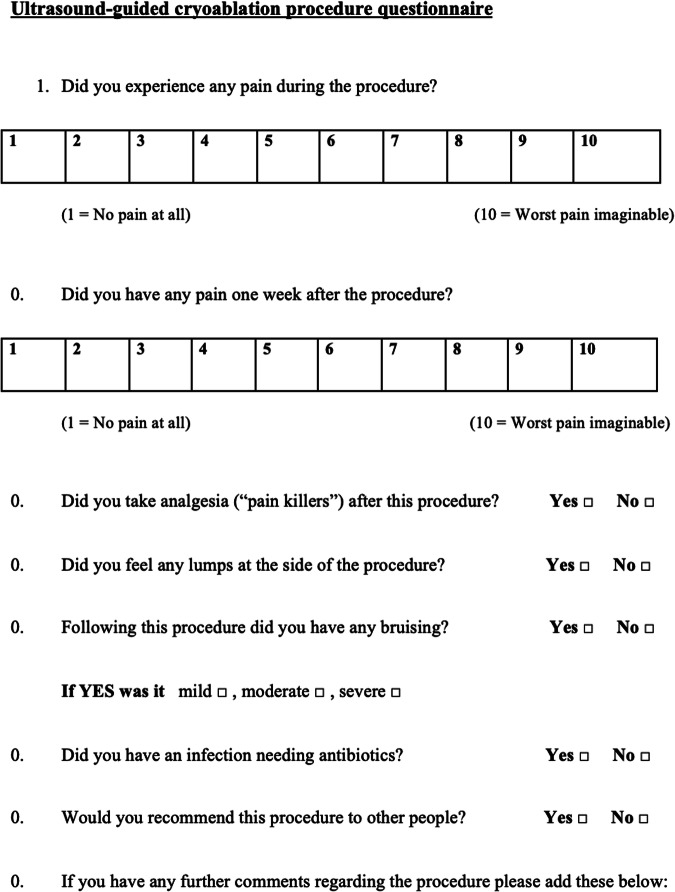
Ultrasound-guided cryoablation procedure questionnaire

Within 10 days from surgical resection, patients received a satisfaction questionnaire (Fig. 2). Patients enrolled in the cryo-group were asked to rate their level of pain during the procedure and one week after the procedure on a scale of 1–10. They were asked if they felt any swelling or noticed any hematoma in the treated area after cryoablation. They were also asked about their need for analgesics after the procedure or antibiotics in case of wound infection. Finally, patients were asked about the esthetic outcome and whether they would recommend the treatment they received to other patients. Patients belonging to the control group were asked to rate, on a scale of 1–10, postoperative pain in the treated area, whether they had any postoperative complications, whether they were satisfied with the final esthetic result of the surgical treatment, and whether they had any postoperative complications.

### Criteria for MRI/CEM evaluation of response to cryoablation and data presentation

Objective response in the cryo-group was defined as complete response or partial response according to response evaluation criteria in solid tumors (RECIST 1.1) criteria [10], based on measurable disease before and after cryoablation, using imaging methods such as MRI and CEM. Due to the small sample size, only a descriptive analysis of the data was possible.

## Results

### Population and cryoablation acceptance rate

From July 2022 to January 2023, we enrolled 20 patients. Ten of them, with a cryo-feasible cancer location, were sent to the cryo-group and ten to the control group. Of the eleven patients screened for enrollment in the cryo-group, only one refused enrollment because the patient chose to be treated at another hospital. Thus, patient acceptance of cryoablation was 10/11 (91%). All the ten patients screened for the control group were enrolled. Patient population and BC characteristics are summarized in Table 2.

**Table 2:** Patient population and BC characteristics

	Cryo-group	Control-group
Age, years, mean (range)	65 (47–80)	62 (39–84)
Menopausal/regular menstrual cycle	9/1	6/4
Tumor size, mm, mean (range)	9.9 (6–18)	10.5 (6–13)
Histopathology	8 No special type 2 Invasive lobular carcinoma	7 No special type 1 Invasive lobular carcinoma 1 Apocrine carcinoma 1 Mucinous carcinoma
Grading	1 G1 8 G2 1 G3	2 G1 4 G2 4 G3
Molecular subtype	4 Luminal A 5 Luminal B 1 HER2-enriched	4 Luminal A 3 Luminal B 3 HER2-enriched

*HER2* Human epidermal growth factor receptor 2

### Surgical procedures

All 20 patients underwent surgical resection within 21 days of enrollment. Surgery was quadrantectomy in 19 tumors and mastectomy in one control patient who was particularly small-breasted. The average weight of the surgical specimens in the cryo-group was 45.4 g (range 15–110 g) and that of the control group was 36.7 g (range 6–116 g). Axillary sentinel lymph node dissection consisted of the removal of 1–4 lymph nodes in 15/20 cases (75%). In the remaining five cases, the number of lymph nodes retrieved ranged from 6 to 8. However, in these cases, the lymph nodes exceeding the number of four were detected only by histology and measured less than 2 mm in diameter. After sentinel lymph node biopsy, no axillary metastases were found on histopathologic analysis.

### Histopathological analysis

Surgical specimens were analyzed by two breast pathologists with more than 15 years of experience at the Department of Pathology of our hospital. All ten patients enrolled in the control group had a biopsy diagnosis of invasive BC, confirmed on the surgical sample. There were seven invasive no special type BCs, one lobular, one apocrine, and one mucinous BC (Table 2). The size of the surgical samples ranged from 6 × 4 × 1 cm^3^ to 10 × 7 × 5 cm^3^.

**Fig. 3:**
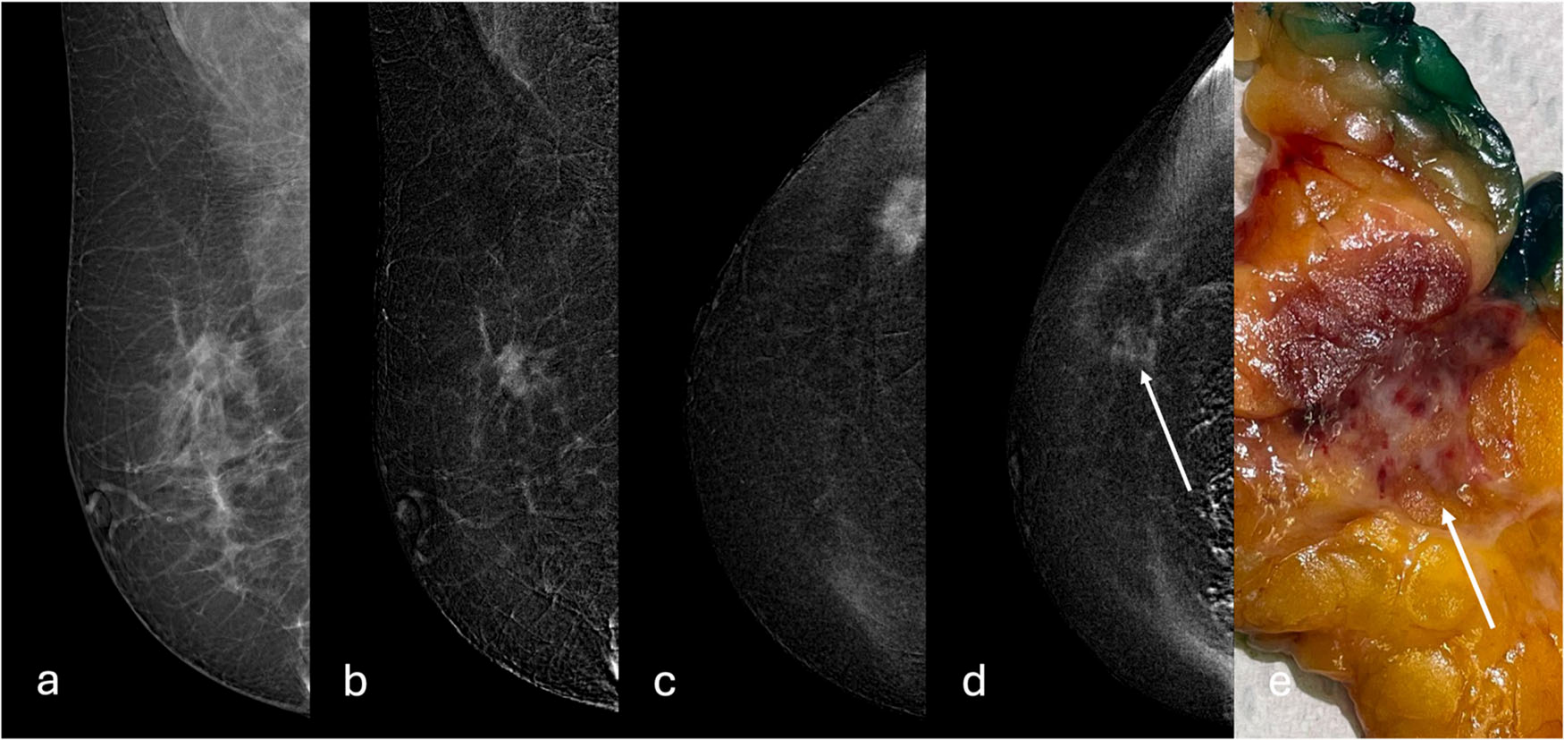
CEM evaluation of cryoablation efficacy and technical success. Pre-cryoablation CEM MLO view of the right breast: **a** low-energy image shows a 14-mm mass with irregular shape and spiculated margins in the upper outer quadrant; **b** MLO and (**c**) CC recombined images show a corresponding mass enhancement; **d** post-cryoablation CC recombined image shows an area of inflammation surrounding the treated area and a 5 mm focus of contrast enhancement in the peripheral zone of the tumor bed (white arrow); and (**e**) the gross specimen shows a homogenous hemorrhagic area with a peripheral white portion (white arrow) that corresponds histologically to a residual component of invasive no special type carcinoma, estrogen receptor 98%, progesterone receptor 30%, human epidermal growth factor receptor 2 (HER2) 0, proliferation index (Ki-67) 24%

Of the ten patients enrolled in the cryo-group, eight had a biopsy-proven diagnosis of invasive no special type BC, and two had an invasive lobular carcinoma (Table 2). The interventional radiologist was always able to complete the procedure, and steatonecrosis was observed in ten out of ten surgical specimens, with complete tumor ablation in nine out of ten patients. The postoperative specimen size ranged from 6 × 4 × 2 cm^3^ to 9 × 8.5 × 3 cm^3^. On gross examination, the cryoablation-treated tumor area appeared as a rounded hemorrhagic lesion surrounded by a rim of steatonecrosis. For each case, the entire lesion was sampled for histology. Microscopic examination confirmed the presence of hemorrhagic areas with neoformed and congested blood vessels, surrounded by steatonecrosis with foamy macrophages and various degrees of inflammation both acute with neutrophilic granulocytes and chronic with lymphomononuclear cells. Additional foci of steatonecrosis were detected in the context of the hemorrhagic areas. Residual invasive tumors were present only in one out of ten cases. The amount of residual disease was 10% of the tumor bed, located in a peripheral location (Fig. 3). Thus, it was probably due to a failed targeting.

### Pre- and post-cryoablation contrast-enhanced imaging

Of the ten patients in the cryo-group, prior to cryoablation, five underwent MRI and five CEM. All tumors showed “masslike” enhancement on MRI and CEM.

**Fig. 4:**
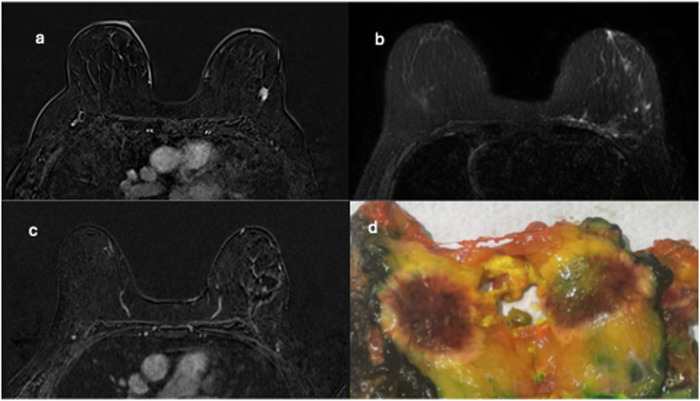
MRI assessment of cryoablation efficacy and technical success. **a** Pre-cryoablation MRI: axial contrast-enhancedT1-weighted subtracted image shows a 12-mm mass lesion with irregular margins in the upper outer quadrant of the left breast. **b** Post-cryoablation MRI: axial unenhanced T2-weighted fat-suppressed image shows minimal edematous imbibition at the site of cryoablation, consistent with post-procedural inflammation. **c** Axial contrast-enhanced T1-weighted subtracted image shows a peripheral inflammatory enhancement of the treated area. **d** The corresponding gross specimen shows a central brown hemorrhagic area surrounded by a yellow halo

**Fig. 5:**
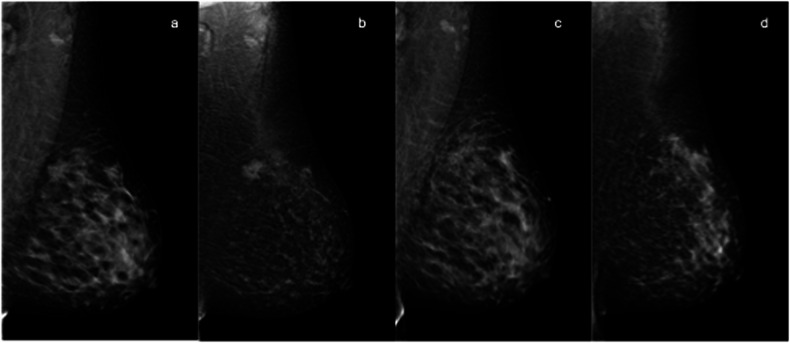
CEM evaluation of cryoablation efficacy and technical success. Pre-cryoablation CEM MLO views of the left breast: **a** low-energy image shows a 16-mm mass with spiculated margins between the upper quadrants; **b** recombined image shows a corresponding mass enhancement. Post-cryoablation CEM MLO views of the left breast: **c** low-energy image shows an area of parenchymal distortion in the upper quadrants and (**d**) recombined image shows no enhancement at the site of the lesion treated with cryoablation

The post-procedural status was evaluated with MRI in five patients, with CEM in four patients, and with ultrasound in one patient who refused both MRI and CEM. MRI or CEM correctly predicted cryoablation technical efficacy in nine out of nine cases (complete cryoablation in eight patients and incomplete cryoablation in one patient). In the eight cases of histopathological complete tumor ablation, no residual enhancement was observed on MRI or CEM after the procedure, resulting in a negative predictive value of 8/8 (100%). The only case of incomplete cryoablation showed a peripheral 4-mm enhancement on CEM, corresponding to the residual component of invasive no special type BC seen in the surgical specimen. On pre-cryoablation mammography and ultrasound, this lesion was irregularly shaped, with spiculated margins, 14 mm in size, with a homogeneous enhancement on CEM (Fig. 3). Examples of pre- and post-procedural MRI and CEM in the case of technical success are shown in Figs. 4 and 5, respectively.

### Satisfaction questionnaire

Pain intensity during cryoablation was rated, on a scale from 1 to 10, as follows: 1 by 2/10 (20%) of patients, 2 by 3/10 (30%), 3 by 3/10 (30%), 4 by 1/10 (10%), and 7 by 1/10 (10%) (resulting in a median pain rating of 3, *i.e*., mild pain). Although there was some swelling in the treated area after the procedure, due to the effect of the ice ball, it did not cause any discomfort. In particular, one week after the procedure, 8/10 patients (80%) rated the pain as 2, and 2/10 patients (20%) rated it as 1. Regarding the cosmetic results after cryoablation, on a scale from 1 to 10, 5/10 patients (50%) rated it 8, 2/10 (20%) rated it 9, and 3/10 (30%) rated it 10. For what concerns minor complications, two patients reported a small post-ablative hematoma (about 4 cm in size) that resolved within one week.

In the days following the procedure, 5/10 patients (50%) reported the use of analgesics, as suggested by the physician. There were no reports of wound infection or other complications.

A satisfaction questionnaire was also administered to patients in the control group after surgery. In the treated area a pain rate of 3 was assigned by 4/10 patients (40%), 5 by 1/10 (10%), 6 by 2/10 (20%), and 7 by 3/10 (30%), resulting in a median pain rating of 5, *i.e*., moderate pain. Patients in the control group reported higher pain scores than those in the cryo group, even though none of the 20 patients had any significant postoperative complications.

## Discussion

The use of cryoablation for breast disease began in 1987 for benign breast lesions (*i.e*., fibroadenomas) and rapidly increased after 2000 [11]. In recent years, cryoablation has become a viable alternative to surgery for the treatment of selected BC patients who are ineligible for surgical resection [12]. Although BC cryoablation has to be still considered in the experimental phase, complete tumor cell death within the ablated zone has been observed in the majority of cases [12, 13].

In our pilot study, we screened 21 patients with an early-stage BC (T1 N0) scheduled for breast surgery. Of eleven patients selected for enrollment in the cryo-group, ten were accepted; thus, patient acceptance of cryoablation in our pilot experience was excellent (91%). To the best of our knowledge, there is no data in the current literature on the acceptance rate of BC cryoablation. So, even considering the very small sample size, this data plays in favor of this approach from the patient’s viewpoint.

Manenti et al [13] reported on 15 women with early-stage BC (lesion diameter of 4–12 mm): complete necrosis after cryoablation was obtained in 14 of them according to surgical specimens after lumpectomy and documented also by postprocedural MRI. The one case with residual disease (at histopathology and MRI) was attributed to incorrect probe placement. A multicenter study performed by Fine et al [14] on 194 patients with unifocal invasive ductal carcinoma of approximately 15 mm treated with cryoablation reported a recurrence rate of 2.06% on the same side of the BC, during a period of about 35 months. These studies concluded that cryoablation is an effective and safe treatment. Consistent with these literature data, in our pilot study where early-stage BC (median lesion size 9.5 mm) was enrolled, a complete tumor ablation was observed in nine out of ten patients (90%). In the remaining case, postoperative histological analysis of the surgical specimen showed a residual disease (10% of the tumor bed), also visible as a focal enhancement on CEM imaging. The lesion met all our eligibility criteria, although its irregular shape probably led to incorrect treatment planning and lack of technical success. As previously mentioned, the peripheral location of the post-cryoablation enhancement suggests a failed targeting as the cause of the residual BC component (Fig. 3).

Two clinical trials are currently ongoing: the ICE3 trial [15] and the FROST trial [16], which are recruiting women aged 50 years or older, with hormone receptor-positive and HER2-negative early-stage BC. The aim of these trials is to evaluate the efficacy of cryoablation, not followed by surgery, and its impact on local and five-year overall recurrence. Although interim results from the ICE3 trial have been published, final results are expected in December 2024. Differently, in our study design, the selection of patient age and BC molecular subtypes was not as strict requirement. Therefore, since we enrolled early BCs with more heterogeneous biological characteristics and prognosis, we decided to perform surgery after cryoablation, also to be sure about possible residual disease not detected at MRI or CEM.

Regarding safety and complications associated with cryoablation, a systematic review and meta-analysis by van de Voort et al [2] investigated whether thermal ablation is an effective method for the treatment of early BC and analyzed various minimally invasive techniques. The selected studies on cryoablation, including 397 patients with BC up to 2 cm in size, reported a pooled complete response rate of 82–89%. In addition, cryoablation had the lowest number of associated complications among minimally invasive techniques. The authors concluded that cryoablation is a promising technique as an alternative to surgical resection without compromising current treatment efficacy or safety. In the aforementioned study by Fine et al [14], post-cryoablation adverse events were reported as mild in 18.4% and moderate in 2.4% of patients. No serious device-related adverse events were reported. More than 95% of patients and 98% of clinicians surveyed reported being satisfied with the cosmetic results achieved. In our study, only two of the ten patients who underwent cryoablation reported minimal skin redness and edema and no serious adverse effect was reported.

Literature on patient satisfaction after ultrasound-guided cryoablation is lacking. However, the review on cryoablation of fibroadenomas by Niu et al [17] reported that existing studies have shown patient satisfaction rates as high as 91–100%. Similarly, in our study, all enrolled women responded positively to the satisfaction questionnaire. All patients in the cryo-group rated the overall esthetic results of cryoablation as excellent and would recommend the procedure as an alternative to surgery due to the lack of permanent skin outcomes (surgical scar). Thus, the cryo-group would recommend this minimally invasive treatment as an alternative to surgery in the future, considering the good cosmetic results and the lower psychological impact.

In addition, the outpatient management of the cryoablation procedure, performed under local anesthesia and without the need for sedation and hospitalization, was well accepted by our patients. It also eliminates the need for preprocedural hospitalization and waiting-time, resulting in an extremely fast turnaround time and cost-saving.

A specific feature of cryoablation, which distinguishes it from all other minimally-invasive ablative techniques including radiofrequency, microwaves, laser, and high-intensity focused ultrasound, is the use of hypothermia instead of hyperthermia. Whereas hyperthermia melts cell membranes and causes protein denaturation, in contrast, cryoablation, using cooling, leaves tumor proteins and tumor-associated antigens intact. In particular, the cooling phase results in coagulative necrosis, which mainly affects the central tissues in the ablation zone, while peripheral tissues undergo delayed apoptosis due to mitochondrial damage [8, 18]. During the thawing phase, tumor cells within the ice ball release tumor antigens, nuclear proteins, and pro-inflammatory cytokines. These signals attract macrophages, natural killer cells, and granulocytes, stimulating the immune response and resulting in the release of antigen-presenting cells that reach the cryoablated tissue [8, 19]. Current evidence suggests that the best way to enhance the immune response would be to block tumor checkpoints, leading to the identification of new cryoablative self-antigens by the immune system [8]. Some pilot studies have demonstrated the synergistic effects of this combined approach with encouraging results [20], and ongoing clinical trials are proposing cryoablation combined with immune checkpoint inhibitors (NCT02833233, NCT03546686, and NCT04249167, http://www.ClinicalTrials.gov). Another special effect, the so-called “abscopal effect”, has also been hypothesized to occur following cryoablation. This effect results in a reduction of distant metastatic lesions [21], and the cause would be the release of tumor-specific antigens that the immune system uses to trigger a specific response toward the tumor [8]. Thus, if cryoablation of BC can induce an antitumor immune response capable of reducing both local and distant disease, and the combination of cryoablation with immunotherapy can be the key to immune treatment for neoplastic lesions, then this approach may be potentially superior to other ablative technologies and become an essential additional tool in the oncological treatment of BC.

Following cryoablation, it is important to monitor procedural efficacy or even the presence of residual disease. Currently, MRI is considered the most accurate modality to assess the effectiveness of the procedure [8]. Manenti et al [22] compared the efficacy of radiofrequency ablation *versus* cryoablation in the treatment of early-stage BC. MRI at one and four weeks after each of the two procedures, showed altered signal intensity and mild peripheral enhancement in the ablated areas. This study confirmed a high negative predictive value of MRI at both one- and four-weeks follow-up after cryoablation, and a strong correlation between enhancement volume on MRI and residual disease on pathology (*r* = 0.896, *p* < 0.0001). Simmons et al [23] conducted a phase II, nonrandomized study evaluating the success of cryoablation of BC, with surgical resection after cryoablation. Successful ablation of the targeted lesion was observed in 80 of 87 cancers (92%) and the negative predictive value of MRI to determine residual invasive BC or ductal carcinoma *in situ* was 81.2%. This supports the potential use of ultrasound-guided cryoablation in selected patients with invasive BC and the high predictive value of MRI to assess cryoablation efficacy.

To our knowledge, our study is one of the first to evaluate the efficacy of cryoablation using MRI or CEM. In this pilot experience, CEM showed excellent results in predicting the outcome of cryoablation and the presence of residual disease after the procedure (see Figs. 3 and 5). A recent review by Corines et al [24] highlighted the value of CEM as a valid imaging technique to evaluate cryoablation outcomes due to its high sensitivity for residual cancer detection and advantages in positive predictive value, time, cost, eligibility, and accessibility compared to contrast-enhanced MRI.

Regarding the control group, nine patients underwent quadrantectomy and one patient underwent mastectomy due to a particularly small breast. None of the ten patients in the control group had relevant post-operative complications, which is consistent with the literature [4], and satisfaction questionnaires reported satisfactory results.

Finally, it is important to acknowledge the limitations of our study. First, the very small sample size, since our was a single-center study with a very small number of patients. Second, the use of contrast-enhanced imaging (CEM and MRI) to evaluate the technical efficacy of cryoablation has excluded some patients (for instance patients with severe allergy to contrast agents, patients with renal insufficiency, and pregnant women). Nevertheless, our results are encouraging and pave the way for future research adopting cryoablation as a non-surgical alternative to treat early-stage BC, as well as to strengthen the predictive value of MRI and CEM for cryoablation outcomes.

In conclusion, our pilot experience showed that ultrasound-guided cryoablation is a safe, effective, and well-tolerated procedure. In addition, both MRI and CEM were able to predict the efficacy of the procedure or even residual disease after cryoablation. The high patient acceptance, the absence of serious complications, and the excellent post-procedural esthetic results support the value of cryoablation as a future alternative to surgery in well-selected patients with early-stage BC.

## Data Availability

The data that support the findings of this study are available from the corresponding author, FG, upon reasonable request.

## Abbreviations

BC: Breast cancer
CEM: Contrast-enhanced mammography
MRI: Magnetic resonance imaging

## References

[1] Van de Voort EMF, Struik GM, Koppert LB et al (2021) Treatment of early-stage breast cancer with percutaneous thermal ablation, an open-label randomised phase 2 screening trial: rationale and design of the THERMAC trial. BMJ Open 11:e052992. 10.1136/bmjopen-2021-052992 34489297 10.1136/bmjopen-2021-052992 PMC8422491

[2] Van de Voort EMF, Struik GM, Birnie E et al (2021) Thermal ablation as an alternative for surgical resection of small (≤ 2 cm) breast cancers: a meta-analysis. Clin Breast Cancer 21:e715–e730. 10.1016/j.clbc.2021.03.004 33840627 10.1016/j.clbc.2021.03.004

[3] Pediconi F, Marzocca F, Cavallo Marincola B, Napoli A (2018) MRI-guided treatment in the breast. J Magn Reson Imaging 48:1479–1488. 10.1002/jmri.26282 30318672 10.1002/jmri.26282

[4] Al-Hilli Z, Wilkerson A (2021) Breast surgery: management of postoperative complications following operations for breast cancer. Surg Clin North Am 101:845–863. 10.1016/j.suc.2021.06.014 34537147 10.1016/j.suc.2021.06.014

[5] Khan SY, Snitman A, Habrawi Z et al (2023) The role of cryoablation in breast cancer beyond the oncologic control: COST and breast-Q patient-reported outcomes. Ann Surg Oncol 30:1029–1037. 10.1245/s10434-022-12570-5 36171531 10.1245/s10434-022-12570-5

[6] Niu L, Zhou L, Xu K (2012) Cryosurgery of breast cancer. Gland Surg 1:111–118. 10.3978/j.issn.2227-684X.2012.08.01 25083433 10.3978/j.issn.2227-684X.2012.08.01 PMC4115688

[7] Roknsharifi S, Wattamwar K, Fishman MDC et al (2021) Image-guided microinvasive percutaneous treatment of breast lesions: Where do we stand? Radiographics 41:945–966. 10.1148/rg.2021200156 34197250 10.1148/rg.2021200156

[8] Galati F, Marra A, Cicciarelli F et al (2024) Cryoablation for the treatment of breast cancer: immunological implications and future perspectives. Utopia or reality? Radiol Med 129:222–228. 10.1007/s11547-024-01769-z 38296892 10.1007/s11547-024-01769-z PMC10879305

[9] Olagunju A, Forsman T, Ward RC (2022) An update on the use of cryoablation and immunotherapy for breast cancer. Front Immunol 13:1026475. 10.3389/fimmu.2022.1026475 36389815 10.3389/fimmu.2022.1026475 PMC9647043

[10] Eisenhauer EA, Therasse P, Bogaerts J et al (2009) New response evaluation criteria in solid tumours: revised RECIST guideline (version 1.1). Eur J Cancer.; 45:228–247. 10.1016/j.ejca.2008.10.026 19097774 10.1016/j.ejca.2008.10.026

[11] Rand RW, Rand RP, Eggerding F et al (1987) Cryolumpectomy for carcinoma of the breast. Surg Gynecol Obstet 165:392–396 2823400

[12] Graña-López L, Pérez-Ramos T, Villares A, Vázquez-Caruncho M (2022) Cryoablation of breast lesions: our experience. Radiologia 64:49–53. 10.1016/j.rxeng.2021.09.002 35428468 10.1016/j.rxeng.2021.09.002

[13] Manenti G, Perretta T, Gaspari E et al (2011) Percutaneous local ablation of unifocal subclinical breast cancer: clinical experience and preliminary results of cryotherapy. Eur Radiol 21:2344–2353. 10.1007/s00330-011-2179-2 21681574 10.1007/s00330-011-2179-2

[14] Fine RE, Gilmore RC, Dietz JR et al (2021) Cryoablation without excision for low-risk early-stage breast cancer: 3-year interim analysis of ipsilateral breast tumor recurrence in the ICE3 trial. Ann Surg Oncol 28:5525–5534. 10.1245/s10434-021-10501-4 34392462 10.1245/s10434-021-10501-4

[15] Cryoablation of low risk small breast cancer-Ice3 Trial (2024) ClinicalTrials.gov ID NCT02200705. https://clinicaltrials.gov/study/NCT02200705. Updated 20 Apr 2018. Accessed Apr 2024

[16] Cryoablation of small breast tumors in early stage breast cancer (FROST) (2024) ClinicalTrials.gov ID NCT01992250. https://clinicaltrials.gov/study/NCT01992250. Updated 20 Apr 2018. Accessed Apr 2024

[17] Niu L, Wu B, Xu K (2012) Cryosurgery for breast fibroadenomas. Gland Surg 1:128–131. 10.3978/j.issn.2227-684X.2012.08.02 25083435 10.3978/j.issn.2227-684X.2012.08.02 PMC4115690

[18] Mehta A, Oklu R, Sheth RA (2016) Thermal ablative therapies and immune checkpoint modulation: Can locoregional approaches effect a systemic response? Gastroenterol Res Pract 2016:9251375. 10.1155/2016/9251375 27051417 10.1155/2016/9251375 PMC4802022

[19] Abdo J, Cornell DL, Mittal SK, Agrawal DK (2018) Immunotherapy plus cryotherapy: potential augmented abscopal effect for advanced cancers. Front Oncol 8:85. 10.3389/fonc.2018.00085 29644213 10.3389/fonc.2018.00085 PMC5882833

[20] McArthur HL, Diab A, Page DB et al (2016) A pilot study of preoperative single-dose ipilimumab and/or cryoablation in women with early stage breast cancer with comprehensive immune profiling. Clin Cancer Res 22:5729–5737. 10.1158/1078-0432.CCR-16-0190 27566765 10.1158/1078-0432.CCR-16-0190 PMC5161031

[21] Regen-Tuero HC, Ward RC, Sikov WM, Littrup PJ (2021) Cryoablation and immunotherapy for breast cancer: overview and rationale for combined therapy. Radiol Imaging Cancer 3:e200134. 10.1148/rycan.2021200134 33817653 10.1148/rycan.2021200134 PMC8011444

[22] Manenti G, Scarano AL, Pistolese CA et al (2013) Subclinical breast cancer: minimally invasive approaches. Our experience with percutaneous radiofrequency ablation vs. cryotherapy. Breast Care 8:356–360. 10.1159/000355707 24415989 10.1159/000355707 PMC3861851

[23] Simmons RM, Ballman KV, Cox C et al (2016) A phase II trial exploring the success of cryoablation therapy in the treatment of invasive breast carcinoma: Results from ACOSOG (Alliance) Z1072. Ann Surg Oncol 23:2438–2445. 10.1245/s10434-016-5275-3 27221361 10.1245/s10434-016-5275-3 PMC5433250

[24] Corines MJ, Sogani J, Hogan MP et al (2024) The role of contrast-enhanced mammography after cryoablation of breast cancer. AJR Am J Roentgenol 222:e2330250. 10.2214/AJR.23.30250 38019473 10.2214/AJR.23.30250

